# Fostering Research Integrity through Training: The training materials of four EU-funded projects through the lens of pedagogical underpinnings and three taxonomies of learning

**DOI:** 10.12688/openreseurope.20826.1

**Published:** 2025-09-26

**Authors:** Erika Löfström, Tom Lindemann, Fenneke Blom, Natalie Evans, Mariette van den Hoven, Giulia Inguaggiato, Panagiotis Kavouras, Dirk Lanzerath, Julia Priess-Buchheit, Rita Santos, Anu Tammeleht, P. J. Wall, Linda Zollitsch

**Affiliations:** 1 Department of Education, University of Helsinki, Helsinki, Finland; 2 Luxembourg Agency for Research Integrity, Esch-sur-Alzette, Luxembourg; 3 Research Support, Research Policy Office, Amsterdam UMC, Amsterdam, The Netherlands; 4 Amsterdam UMC location Vrije Universiteit Amsterdam, Ethics Law and Humanities, Boelelaan 1117, Amsterdam, The Netherlands; 5 Centre for Medical Ethics, University of Oslo, Oslo, Norway; 6 German Reference Centre for Ethics in the Life Sciences (DRZE), University of Bonn, Bonn, Germany; 7 Christian-Albrechts-Universität zu Kiel, Kiel, Germany; 8 University of Tartu, Tartu, Estonia; 9 ADAPT Centre, Technological University Dublin, Dublin, Leinster, Ireland

**Keywords:** Research integrity, research ethics, training, learning objectives, learning taxonomies

## Abstract

**Background:**

This article provides an analysis of the pedagogical underpinnings that characterise the research integrity (RI) training approaches and materials developed by four European Union - funded projects. The approaches taken by these projects include a dialogical approach, an empowerment approach, a virtue ethics approach, and a constructivist case-based approach. We analysed the materials/trainings created in the projects. In doing so, we propose how to make use of a structured way of working with learning objectives, in order to ensure that research integrity training meets the needs of its target groups.

**Methods:**

We applied qualitative, deductive content analysis in which we analysed the learning objectives stated in the training schemes of the four projects. A total of 46 learning objectives were analysed using three learning taxonomies, namely the revised Bloom's Taxonomy, the SOLO Taxonomy, and the Taxonomy of Significant Learning.

**Results:**

The results show that the four RI trainings make use of either a constructivist or a socio-constructivist understanding of learning, implement activating and engaging learning activities, and emphasise high-order learning objectives.

**Conclusions:**

The analysis suggests that taxonomies are applicable to various pedagogical underpinnings and can help trainers to communicate the objectives of training and learners to relate their expectations to the objectives. We identify implications for training design and suggest recommendations for training developers. It is advisable to pay attention to learning objectives dedicated to foster the higher levels of learning and understanding. Less commonly applied taxonomies in the context of integrity training, such as the Taxonomy of Significant Learning can help to identify relevant learning objectives both for trainers as well as learners. Research cultures and disciplinary differences are generally not spelled out at the level of learning objectives highlighting the need to consider these explicitly in training implementation.

## Introduction

There is consensus among academics that fostering a culture of integrity requires formal training ( Bertram-Gallant, 2011; Forsberg *et al*., 2018; Mejlgaard *et al*., 2020; Resnik & Shamoo, 2011). The provision of research integrity (RI) training is also emphasised by the European Code of Conduct for Research Integrity ( ALLEA, 2017; ALLEA, 2023) and many national codes ( Godecharle *et al.*, 2013). RI training aims to develop knowledge, skills, values, attitudes, and behaviour individually and collectively in relation to the principles and standards that ensure the validity and trustworthiness of research ( Kalichman, 2014; Kalichman & Plemmons, 2007; Pizzolato & Dierickx, 2021; WCRIF, 2017). Several studies suggest that RI education should start early and continue throughout the research career ( Johansen *et al*., 2022; Labib *et al*., 2023; Pizzolato & Dierickx, 2021; Zucchero, 2008).

Furthermore, it has been suggested that integrating RI content into discipline-specific curricula ensures the integration of RI content into regular teaching, rather than being limited to a single training session that is only loosely connected to participants' overall learning ( Zucchero, 2008). Research has identified pedagogically sound and engaging teaching methods and activities for teaching RI ( Berling *et al*., 2019; Kalichman *et al*., 2022; Katsarov *et al*., 2022; Löfström, 2016; Nonis & Swift, 2001; Tammeleht *et al.*, 2019; Tammeleht *et al.*, 2021; Tammeleht *et al.*, 2022). These include methods such as case studies, collaboration, role plays and real-life simulations. Recently, research has also aimed at identifying how to measure RI training effectiveness ( Tammeleht & Löfström, 2023; Tammeleht *et al*., 2024; Tammeleht & Löfström, 2025a; Tammeleht & Löfström, 2025b; van Loon *et al*., 2023). This article provides an analysis of the pedagogical underpinnings that characterise the training approaches and materials of four European Union - funded projects, as well as examples of how to make use of a structured way of working with learning objectives, in order to ensure that RI training meets the needs of its target groups.

In the past decade, the European Commission has signalled how RI is a priority, and funding has been made available to develop RI training programs. Funded projects have different theoretical and conceptual backgrounds, learning objectives, and target groups. Analysing these projects provides educators an understanding of the differences between them and the areas where they complement each other. Between 2016 and 2022, four European projects, namely Path2Integrity, INTEGRITY, VIRT²UE and ENERI developed RI trainings targeting a variety of audiences, including high school students, tertiary students, early career researchers, senior researchers, research administrators, experts, and research ethics and RI committee members. All four projects developed innovative and engaging approaches to facilitate learning amongst their target groups. Whilst other projects have also developed RI training within The Commission’s Horizon scheme, we focus on these four relatively early projects funded under Horizon 2020, because of their recognised impact in the European Research Area and their range of relevant target groups ( European Commission, 2024).

The aim of this article was to conduct an analysis of the approaches to RI training developed and promoted by these four projects funded by the European Union. In doing so, we analyse the project’s materials/trainings using three learning taxonomies, namely Bloom's Taxonomy (original Bloom *et al*., 1956; revised version, Krathwohl, 2002), the SOLO Taxonomy ( Biggs, 1996; Biggs, 1999), and the Taxonomy of Significant Learning ( Dee Fink, 2003). Through the analysis, this paper provides a structured overview of the types of learning objectives that were promoted through the four projects. We believe this to be valuable in identifying how existing materials and trainings already support RI learning, but also where gaps still exist in terms of support for reaching higher-order RI knowledge and skills. At the same time, this article builds on the work of van den Hoven *et al*. (2023) who compare the learning objectives of INTEGRITY, Path2Integrity and a pre-final version of VIRT²UE to demonstrate the comparative value of taxonomic analysis using a modified version of the assessment-focused Kirkpatrick model, namely the taxonomy for RI training (TRIT) (2023).

In contrast, the three learning taxonomies applied in this study focus on what the learner can do with the knowledge (Bloom’s taxonomy, Bloom *et al*., 1956; Krathwohl, 2002), the levels of understanding (the SOLO taxonomy, Biggs, 1996; Biggs, 1999; Biggs & Tang, 2007), and the affective dimension (The Taxonomy of Significant Learning, Dee Fink, 2003). More precisely, Bloom's widely used taxonomy provides a categorisation of learning objectives based on cognitive complexity. Every stage of Bloom's Taxonomy, namely Remembering, Understanding, Applying, Analysing, Evaluating and Creating, represents a distinct cognitive process and reflects increasing complexity of learning. The original version ( Bloom *et al*., 1956) was later revised for improved systematics ( Krathwohl, 2002). There have been various attempts to revise the taxonomy, and in this process, it has become more focused on a cognitive dimension of learning (as opposed to an affective dimension) ( O’Neill & Murphy, 2010). In a similar vein, focussing on a cognitive dimension of learning, SOLO ( *Structure of Observed Learning Outcomes*) was developed especially for setting goals and assessing learning in tertiary education context. This approach focuses on levels of understanding, namely pre-structural, uni-structural, multi-structural, relational and extended abstract levels of understanding ( Biggs, 1996; Biggs, 1999). In learning at the tertiary level and beyond, it is desirable that learners not only learn isolated bits and pieces of knowledge (multi-structural level) but are able to make connection between the pieces of information (relational level) and connect extended abstract ‘chunks’ of knowledge with each other to form holistic understanding of a given theme ( Biggs & Tang, 2007). The Taxonomy of Significant Learning ( Dee Fink, 2003) is not hierarchical in the same way as the other two, however, it builds on Blooms’ taxonomy re-introducing a long-neglected affective component into the discussion. It identifies foundational knowledge, application, integration, a human dimension, caring, and learning to learn competencies ( Dee Fink, 2003).

We framed our research question as follows: *How are different levels of learning supported through the pedagogical approach, learning objectives and materials of contemporary research integrity training?* We envision educational developers, teachers of RI and others who have an interest in the research-based development of RI training to be our readers.

## Four approaches to fostering research integrity

INTEGRITY, Path2Integrity, VIRT²UE and ENERI embrace the concept of learner-centredness as a central pillar of their educational approach but differ in their pedagogical underpinning. The differences are reflected in the foci of their educational practices. This section provides an overview of the four distinct approaches to training.

### A dialogical approach

Path2Integrity starts from the premise that researchers are members of an active scientific community which continuously discusses, reflects and (re-)develops practices that together constitute responsible conduct of research. Moreover, the project recognizes that the scientific community is embedded into a wider societal community where different actors draw on research results to make evidence-based or evidence-informed decisions. Thus, being a responsible researcher, student or citizen using research results presupposes acquiring and applying discursive skills to competently and confidently address normative challenges and navigate environments characterized by uncertainty. Path2Integrity aims to support the development of such skills by providing learning units on various research integrity topics that seek to enhance the capacity of learners to engage in rational argumentation, set shared objectives and norms, establish preconditions for dialogue, weigh the pros and cons of different possible courses of action and, in general, become active participants in discourses on responsible conduct of research.

More precisely, the project developed the Path2Integrity learning programme to support conducting “a dialogue on the rejection or acceptance of norms in research integrity” ( Prieß-Buchheit *et al*., 2020, 23). To that end, “Path2Integrity follows a twofold dialogical approach: First, the main goal of the learning programme is to conduct a dialogue and second the programme offers dialogue-based learning methods such as storytelling and role-playing to achieve this goal” ( Hermeking & Priess-Buchheit, 2022; Prieß-Buchheit *et al*., 2020). In this way, the learning programme facilitates interactions among learners and between learners and educators and guides them towards discussing their research practices and the reliability of research results, while considering the particularities and challenges of the research environment and research culture they are currently embedded in. The dialogical approach that underpins Path2Integrity ultimately aims to support researchers, aspiring researchers and research stakeholders in building a common language to openly and constructively discuss research integrity issues, answer questions and develop viable solutions for challenges in accordance key principles and norms governing the research community and responsive to societal needs and concerns.

In addition to general questions of research integrity, the learning programme emphasizes identifying and addressing research issues and solutions and the reliability of research results and its determinants, including systemic aspects. Each learning unit is based on engaging exercises to guide learners to conduct dialogues of the rejection or acceptance of norms in research. This translates into a variety of learning goals covering different subfields of research integrity as conceptualized in the European Code of Conduct for Research Integrity ( ALLEA, 2023), such as research environment, research procedures, safeguards, collaborative work, citation and publication, mentoring, publishing and conflict of interest, and data practices (see www.path2integrity.eu to access the materials). Reflecting the overall premises of Path2Integrity, the learning goals consider both individual, researcher-centred and wider societal perspectives on research integrity and the exercises are designed in a way that both perspectives are represented in dialogues.

Dialogues are a key element of all learning sessions. They are fostered through different interactive tasks that are designed to encourage open-sharing and discussion of viewpoints and opinions ( Priess-Buchheit, 2020, 60). In general, “[a] dialogue is a high-quality interpersonal relationship” (cf. Widdershoven & Solbakk, 2019) and seeks to be embedded in an ideal speech situation (cf. Habermas, 1990, 43–115) in which the respective other learner is recognized as a person, where instrumentalization is renounced and the right of others to hold a differing opinion is taken seriously and where subject roles can be clearly defined (cf. Lorenz, 2005, 189–191). In other words, a dialogue can be conducted when impartial, unconstrained and non-persuasive acts are respected (cf. Gethmann, 2005, 191).

Overall, the Path2Integrity learning programme was designed from a developmental perspective to support societal progress by enabling the different target groups to stand up for and argue in favour of responsible research practices and reliable research results. The project thus identified the development stages of its three different core target groups, namely i) senior high school students, ii) university students, and iii) early career researchers and developed a so-called handbook of instruction for teachers of each of the three groups. In total, the Path2Integrity learning programme encompasses 27 learning units, each of which is described in detail on a learning card. All materials were drafted in an iterative process of drafting, using in teaching, collecting feedback and redrafting in 2019 and 2020 ( Priess-Buchheit, 2020).

### An empowerment approach

INTEGRITY focuses on the *empowerment* of students and early career researchers, meaning that instead of seeking compliance, the project took the building of capacities to detect, reflect and act upon RI issues in academic and research practices as a starting point. The view on empowerment is inspired by Freire’s Pedagogy of the Oppressed and has been developed in the project into a competence profile to design, develop and test novel educational tools. The empowerment approach is based on three core elements. First, empowering students is about gaining control in the context in which one is located; hence the project takes into consideration that students perceive and experience academic integrity and RI issues differently throughout their career. Consequently, INTEGRITY’s vision and approach focused on the development of teaching tools that were tailored to students’ own perceptions and needs concerning academic and RI issues, according to their scholarly level and in their own field of studies, from upper high school levels to early career researchers. This was perceived to be important because academic and RI issues are perceived differently in different disciplines (for example, authorship customs differ from humanities to life sciences). Students need to be able to develop critical thinking and autonomy, that is, a reflective attitude on their own practices and experiences. Therefore, students should be empowered to learn what academic and research integrity entails, following the definitions and principles stated in the European Code of Conduct for Research Integrity ( ALLEA, 2017), while, at the same time, building capacity to identify, critically reflect upon, and avoid questionable academic and research practices and misconduct.

Second, to empower learners, it is important to recognise that academic integrity and research integrity issues often entail grey zones ( Goddiksen *et al*., 2024), where there is no ‘right’ or ‘wrong’ approach or answer. Thus, through different pedagogic approaches envisioned in the developed teaching modules, students are engaged in critical reflections and interactive discussions of the grey zones (e.g. loyalty conflicts; Goddiksen *et al*., 2021).

Finally, empowerment stimulates a pro-active attitude in course participants, helping them to actively address and deal with integrity issues they encounter in their studies or research practices.

Like Path2Integrity, the INTEGRITY project included high school students as a target group. Although this group of participants may have limited experience, it should be empowered in academic integrity and RI research integrity issues both as potential future university students and as future citizens. The underlying premise is that RI values not only support the responsible behaviour that young students would likely perpetuate through their academic life, but are also key in empowering responsible citizens. High school students are at a cognitive and emotional development stage where they are building their own perceptions and opinions regarding the society within which they live. Learning to form opinions in an informed and reflective way is important to recognise how their decisions might impact their academic career and also more widely their role within society ( Bishop & Szobota, 2015; Ike & Anderson, 2018).

### A virtue ethics approach

The VIRT²UE project has designed a train-the-trainer programme for teaching research integrity using a virtue ethics approach. VIRT²UE took the perspective that RI is an aspect of professional competence and ethics, and therefore a virtue ethics approach is particularly appropriate for fostering RI amongst researchers. The virtue approach is guided by Aristotelian ethics and Macintyre’s moral philosophy ( MacIntyre, 1981). Specific advantages of taking a virtue ethics approach to teaching RI have been outlined in detail elsewhere ( Evans *et al*., 2024; Pennock & O'Rourke, 2017). These include: 1) moving away from mere compliance to rules by reflecting on how to apply them to specific questions – through training and practice with real-life complex RI situations; 2) strengthening professional ethics – through reflection on the goals and responsibilities of the profession and the researcher qualities that support them; 3) encouraging virtue cultivation - by explicitly linking scientific virtues to the goods that are internal to the practice (such as proficiency) and the ends of the profession (in this case the advancement of knowledge); 4) fostering a common ethos of science – through the recognition and internalisation of scientific virtues, and awareness of how these relate to the principles and practices of shared normative guidance.

In addition to a virtue ethics approach, the programme foundations include stimulating a common ethos of science through increased knowledge of the principles and practices of the European Code of Conduct for Research Integrity ( ALLEA, 2017), learning by doing (reflecting an Aristotelian approach to moral learning) which is reflected in the design of the training programme for teachers and in the programme’s individual exercises, and learner-centred teaching which is reflected in the programme’s learning goals and in the provision of materials that can be selected according to the target group ( Evans *et al*., 2021; Evans *et al.*, 2024).

The VIRT²UE training is a blended learning programme, consisting of a series of e-learning modules and participatory exercises. The e-learning modules are designed for individual learning and reflection, whereas the participatory exercises allow for group reflection on experiences from practice. The conceptual/theoretical foundations of are reflected in the programme structure and materials in the following ways:

Teaching on “virtues”: The programme follows best teaching practices for the cultivation of virtues, including direct instruction on core virtue concepts and terms ( Baehr, 2013; Berkowitz & Bier, 2007) and the application of knowledge through reflection on one’s own character virtues, particularly in relation to specific morally ambiguous situations ( Baehr, 2013). The VIRT²UE TtT (training-the-trainer) programme therefore incorporates both direct instruction via the e-learning modules and reflection related to specific situations experienced in the participatory exercises.

Stimulating a common ethos of science: The programme develops awareness of, and knowledge about, the principles and practices of the European Code of Conduct for Research Integrity ( ALLEA, 2017). Knowledge of the European Code of Conduct for Research Integrity is developed and tested in the programme’s e-learning modules. Application of the core principles of good scientific conduct (reliability, honesty, respect, and accountability) is, furthermore, incorporated into the programme’s participatory exercises.

Learning by doing: The ‘learning by doing’ approach is reflected at different levels of the VIRT²UE programme. First, trainers experience the programme as participants, before learning how to facilitate the programme themselves. Second, the e-learning modules and participatory exercises incorporate aspects of ‘learning by doing’, particularly group learning through dialogue which is at the core of the participatory exercises. Third, the participants are equipped with structured reflection tools that they can use to support decision making in their own research practice.

Learner-centred approach: The learner-centred approach is reflected in the VIRT²UE TtT programme’s learning goals, modular materials (which can be selected depending on the needs of the target group), and emphasis on trainers applying a learner-centred approach in their own teaching.

### A constructivist case-based approach

Research ethics (RE) and RI both form the foundation of good science. While research ethics addresses the design and conduct of research and the protection of human participants and application of ethical principles to different fields of study, RI addresses responsible conduct in accordance with scientific norms and values. To this end, the *European Network of Research Ethics and Research Integrity* (ENERI) project ( www.eneri.eu) targeted experts on research ethics and integrity, providing materials, training, and networking with a view to bridging research ethics (RE) and RI. Themes that experts deal with associated with RI include good scientific practice, questionable research practices, research misconduct, responsible authorship, peer review, and whistleblowing, to name a few. Themes that experts on RE deal with include, for instance, ethics review, the protection of human rights, safety of trials, informed consent procedures, research with participants unable to consent, and protection of animals. Comprehensive and overlapping issues involving both RI and RE related considerations involve, for example, data management and data protection, open data sharing, open access, transparency, fairness, reliability and credibility, and conflict of interest. Developing and sharing good practices around these benefit experts in both domains.

To do this, ENERI facilitated sharing experiences through communication and exchange; training and capacity building and creating an e-Community for experts in research ethics and research integrity. ENERI involved existing networks, projects, and infrastructures, and connected RE and RI experts, such as RI officers and members of RE committees, through stakeholder workshops, training initiatives and by building an integrated network of RE and RI experts.

The ENERI Classroom ( https://classroom.eneri.eu/) is a self-study online platform building on a constructivist view of learning that recognises the importance of learners’ prior knowledge, and learner agency, in the construction of new knowledge. The ENERI Classroom learning objectives are outlined for each theme, the content is structured along recurring rubrics for clarity and cognitive support, and there are activating case tasks through which the learner progresses step-by-step scaffolded by questions and prompts. Constructivism focuses on learning as knowledge construction with an emphasis on structure and cognitive support ( Jonassen, 1997). To learn, an individual must be actively cognitively engaged in the learning process ( Mayer, 2004). Scaffolds involve a variety of techniques that gradually guide learners to greater mastery and insight building on prior steps ( Quintana *et al*., 2004). In the ENERI Classroom, this involves progression through the steps of ethical analysis ( Mustajoki & Mustajoki, 2017). In the context of already expert learners, as in the case of the ENERI Classroom, the role of scaffolding is to widen the scope of perspectives and attune the learner to core questions of research ethics and integrity at the expert level, including leadership and responsibility.

Research has shown that expert-level learners may not benefit from redundant instructional guidance, because of the mental representations of the topic that they already have ( Kalyuga *et al*., 2003). Consequently, the ENERI Classroom provides a limited amount of instructional guidance, mainly a structure for cognitive support, and scaffolded cases for active engagement. Engaging with cases in research ethics or integrity has been shown to improve understanding of topics, support reflection on theory through practice, and enhance understanding of the context (e.g., Burr & King, 2012; Clarkeburn, 2002; Fisher & Kuther, 1997; Rissanen & Löfström, 2014; Tammeleht *et al*., 2019; Tammeleht *et al*., 2022) Constructivist views of learning have been criticized for assuming self-regulation skills and initiative on behalf of the learner, especially young and inexperienced ( Kirschner *et al*., 2006). However, in the case of experts, the project relied on an elaborate level of learners’ own initiative and self-regulation.

## Method

We took a deductive approach, in which we analysed the learning objectives stated in the training schemes of the four projects. We applied qualitative content analysis as follows: We compared each learning objective to the levels of Bloom’s taxonomy (revised version, Anderson & Krathwohl, 2001; Krathwohl, 2002), SOLO taxonomy ( Biggs, 1996; Biggs, 1999), and the Taxonomy of Significant Learning ( Dee Fink, 2003). A learning objective could include more than one level of taxonomic category. To categorise the learning objectives according to Bloom’s revised taxonomy, we used a list of verbs that are associated with different levels ( Anderson & Krathwohl, 2001). Some verbs may be associated with various levels of the taxonomy, such as ‘explain’ (understand and analyse), and ‘relate’ (understand and apply). Consequently, the categorisation had to be done in relation to the idea of learning that the objectives were interpreted to convey rather than automatically assigning a category based on the verb lists. To categorise the learning objectives according to SOLO taxonomy, these were matched with the descriptions of the five SOLO levels, namely pre-structural, unistructural, multi-structural, relational, and extended abstract level of understanding ( Biggs & Tang, 2007). To categorise the learning objectives according to the Taxonomy of Significant Learning, we used a similar verb chart ( Dee Fink, 2003) as for Bloom’s taxonomy. Especially regarding this taxonomy, the learning objectives typically seemed to embrace several categories of the taxonomy at once. This made sense as the taxonomy is not hierarchical, but categories can feature in parallel. A total of 46 learning objectives were analysed.

To ensure reliability of the analysis, all learning objectives according to Bloom’s revised taxonomy, were coded by two individuals, who amongst each other agreed upon the category if disagreement took place. The categorisations applying SOLO and Significant Learning were all done by one author external to the projects, which may have increased objectivity of the analysis, and verified by another (from within a project).

The research did not involve human participants. The data consists of publicised learning objectives of the trainings and materials produced in four European Union -funded projects.

## Results

Before describing the results of the analysis of learning objectives, we summarise key features of the trainings. Theoretical underpinnings of the involved projects relate to the pedagogical theories of Freire’s emancipatory pedagogy and to constructivism, while one of the projects takes its point of departure in ethical theories, namely virtue ethics. The pedagogical approaches involve empowerment, dialogical approach, experiential case-based group learning approach, and a case-based approach ( Table 1). More specifically, the projects utilise storytelling, role-play, constructive agreement, collaboration and reflection, and scaffolded self-study as methods for teaching/learning. Target groups range from upper secondary school to RE and RI experts.

**Table 1.** Theoretical underpinnings, pedagogical approaches, teaching methods, target groups, modalities, and duration.

Project	INTEGRITY	Path2Integrity	VIRT ^2^UE	ENERI Classroom
**Theoretical underpinnings**	Freire’s Pedagogy of the Oppressed	Constructivism	Virtue ethics	Constructivism
**Pedagogical approach**	Empowerment	Dialogical approach	Experiential, case-based group learning processes	Case-based approach
**Method**	Dialogical methods such as case scenarios for group discussion/debate and individual reflection	Dialogical methods such as storytelling, role-play, and coming to an agreement	Both individual and group learning by means of dialogue	Self-study with scaffolded cases
**Ages/researcher stages targeted**	Starting from upper secondary school level (i.e., 17–18 years) to university level (bachelor and PhD students)	Starting from 16 years / secondary school students, bachelor’s and master’s students, early career researchers	Active researchers including Masters’ students, PhD candidates, early career and senior researchers	New and experienced members of research ethics committees and research integrity boards.
**Online/ asynchronous training material**	Online games (Integrity games) & online course for PhD students (MOOC)	Learning units in E-Learning Courses (digital learning cards, handbooks), Blended Learning, international online workshops, P2I campaign with role-models	E-learning modules, supportive materials (reading and videos), and detailed instructions for participants and facilitators of the training available via online teaching guides	Thematically structured online learning units
**Offline/** **face2face/** **synchronous training material**	Modules with gamified case scenarios (board game for high school students)	Learning units in learning cards and handbooks	Participatory exercises via Modules and training manual	Thematically structured learning units
**Duration of asynchronous training**	n/a	90–120 Minutes per learning unit	36 hours ( *4 hours e-learning modules + 7 hours preparatory assignments + 25 hours practicing participatory exercises in own setting*)	n/a
**Duration of synchronous training**	n/a	90–120 Minutes per learning unit	24 hours ( *16 hours first session + 8 hours final session*)	n/a
**Openly available:**	Yes https://h2020integrity.eu/toolkit/	Yes https://www.path2integrity.eu/ri-materials	Yes https://embassy.science/wiki/Guide:Bbe860a3-56a9-45f7-b787-031689729e52	Yes https://classroom.eneri.eu/
**Certificate**	Yes (for PhD course)	Yes	Yes	No

Path2Integrity and VIRT²UE utilise activating, engaging and collaborative methods. The ENERI Classroom is meant for self-study and is less geared towards social and collaborative learning. However, while the emphasis in the other two projects is more on social constructivist ideas of learning, ENERI Classroom implements constructivist ideas through the use of learning objectives supported by structured content and complemented with activities that support engagement with the content. INTEGRITY applies both constructivist and social constructivist-based methods. Two of the projects, namely INTEGRITY and Path2Integrity, offer courses for secondary students in line with the recommendation that training should begin early on ( Tryon, 2000; Zucchero, 2008).

Path2Integrity, VIRT²UE, and ENERI Classroom offer learning units or modules available for use online, offline and/or in face-to-face teaching contexts. Path2Integrity and VIRT²UE offer training manuals or handbooks for facilitators. Path2Integrity offers a wide range of materials that can be used in different formats of training and adapted according to need.

Assessment of learning takes place mainly through reflection, and self-assessment and in the trainings that build on face-to-face or contact teaching, the collaborative processes facilitate feedback from peers. Collaboration allows learners to gain feedback on their ethical decision making ( McCormack & Garvan, 2014). Feedback on the sustainability of one’s ethical reasoning is a key pedagogical element facilitating the learning ( Rissanen & Löfström, 2014). Three projects offer certification as a formal acknowledgement of completed training.

### Path2Integrity learning objectives

The training developed in Path2Integrity targeted secondary school students, bachelor’s and master’s students, and early career researchers. The learning objectives were analysed depending on whether they represent a social ( Table 2) or an individual ( Table 3) dimension, as both were considered relevant in the dialogical approach permeating all objectives.

**Table 2.** Path2Integrity learning objectives with a social dimension.

Learning objective	Bloom’s revised Taxonomy	SOLO Taxonomy	Taxonomy of Significant Learning	Pedagogical underpinnings
1. Explain and justify your argument/ norm/ purpose (when you are asked)	Understanding and evaluating	Relational	Foundational knowledge/ Integration/ Human Dimension/ Caring	**Raise awareness by dialogical approach:** This objective sets the ground for a dialogical setting. Knowledge must be displayed, explained, and justified if someone’s asking for it. This means to deep dive into your own argument/ norm/ purpose and reflect on it. This leads to a better understanding of one’s own knowledge.
2. Accept ambiguity: accept different arguments/ norms/ purposes from the dialogue	Understanding	Relational	Human Dimension/ Application/ Caring	Dialogical approach: Getting involved in the arguments/norms/purposes of others.
3. Reflect on your understanding of what others are saying and consider how you are being understood	Analyzing	Relational	Human Dimension/ Caring/ Integration	Dialogical approach: Making sure to understand others and to be understood by others. Exchange understanding with others.
4. Adjust arguments/ norms/ purposes together with the dialogue group and/or for the target groups	Applying	Extended abstract	Human Dimension/ Integration/ Learning to learn/ Caring	Dialogical approach: Find a common understanding with others.
5. Support the best arguments and solutions arising in the dialogue regardless of whether they are your own or that of others	Analyzing and applying	Relational	Learning to learn/ Caring/ Human Dimension/ Application	Dialogical approach: Don’t stick to an argument/norm/purpose if you know better, don’t let socioeconomic status decide what a good argument is.

**Table 3.** Path2Integrity learning objectives with an individual dimension.

Learning objective	Bloom’s revised Taxonomy	SOLO Taxonomy	Taxonomy of Significant Learning
1. Present all your relevant knowledge and your purpose	Understanding and analyzing	Multistructural	Foundational Knowledge
2. Clarify arguments, solutions, and purposes	Applying	Relational	Application/ Human Dimension/
3. Compare, prioritize arguments, solutions, and purposes	Analyzing	Relational	Integration/ Application
4. Drop arguments, solutions, and purposes, which cannot be logically retraced by others	Aanalyzing, evaluating	Relational	Caring/ Human Dimension/ Application
5. Develop solutions integrating arguments, norms, and purposes from the dialogue	Creating	Extended abstract	Learning to learn/ Integration/ Human Dimension/ Caring

### INTEGRITY learning objectives

The training developed in this project targeted secondary school, undergraduate and graduate level learners. For the secondary school students, a training course with nine modules was developed, including topics that may be part of the high school curriculum (subject to national variation), such as technology, music, animal experimentation, and space exploration. Each module contained a theoretical section to present the concepts that students should learn about research integrity and responsible conduct of research (RCR), followed by a practical activity including dilemma cases and role play for students to work in groups ( Table 4). The combination of theoretical and practical activities was intended to bring about empowerment in two ways, namely through knowledge-acquisition and enacting upon the knowledge. 

**Table 4.** INTEGRITY secondary school learning objectives.

Learning objective	Bloom’s revised Taxonomy	SOLO Taxonomy	Taxonomy of Significant Learning	Pedagogical underpinnings
1. Have basic knowledge about what research entails. This includes basic knowledge about research design, methodology, data, reporting findings	Remembering	Multi-structural	Foundational Knowledge	To empower high school students in RCR first it is critical to provide them with basic knowledge about what research entails, as high school students have little experience in research.
2. Being aware of the relevance and urgency of RCR and research integrity in one’s research project	Understanding & Applying	Relational	Human Dimension/ Integration/ Caring	After acquiring knowledge on the basic principles of research, students are more likely to understand the concepts of RCR and research integrity if they apply them to their own school practices (e.g., when developing a school assignment).
3. Knowing what topics are relevant in current debates and being able to identify and reflect on relevant RCR aspects in any given situation	Understanding, Analysing and Evaluating	Relational	Foundational Knowledge/ Learning to learn/ Human Dimension/ Integration	Fictional case-dilemmas of secondary school life offers an opportunity for students to identify the unethical practices at stake, debate and critically reflect with other students on the best practices to adopt in the different situations. The debating of case-dilemmas stimulates students’ ability to formulate and justify their opinions.
4. Ability to solve basic integrity issues in doing research	Applying	Extended abstract	Application/ Caring	Applying the fundamental concepts of RCR to students’ school activities facilitates solving integrity issues and engaging in ethical practices.
5. Seek and welcome help from others in a disturbing situation	Analysing & Evaluating	Multi-structural	Human Dimension/ Caring/ Learning to learn	Peer learning
6. Being able to communicate in an open manner about RCR issues	Creating	Extended abstract	Caring/ Human Dimension/ Application	After learning about RCR, students are more prompted (empowered) to act responsibly when doing their coursework and raise their voices against unethical actions they observe or experience.

The undergraduate tool covers three themes, namely ‘Drawing on the work of others’ (including plagiarism), ‘Collaboration’, and ‘Collecting, analysing and presenting data’ ( Table 5).

**Table 5.** INTREGRITY undergraduate learning objectives.

Learning objective	Bloom’s revised Taxonomy	SOLO Taxonomy	Taxonomy of Significant Learning	Pedagogical underpinnings
1. Knowledge of the core RI/AI values/principles and how they are applied under each topic	Remembering & Understanding	Relational	Foundational Knowledge/ Application/ Learning to learn	Learning and comprehending the fundamental principles of academic/research integrity and RCR sets the mote so then students relate these principles to their own academic practices.
2. Knowledge of common grey area issues and the reasons why they are “grey”	Remembering & Understanding	Multi-structural	Foundational Knowledge/ Learning to learn	Academic and Research Integrity issues are not “black and white”, and students should learn and be able to identify the grey areas to be better equipped to make ethical decisions.
3. Competences in making decisions in light of multiple values/principles	Analysing & Evaluating	Relational	Foundational Knowledge/ Learning to learn/ Application/ Integration	Case-dilemmas offer an opportunity for students to identify common integrity issues that could arise from their academic work and critically reflect on the ethical conduct. At the same time, students are exposed to grey areas (e.g., morality conflicts) during the decision-making process.
4. Motivation to develop further RI knowledge skills and competences (positive nudging)	Creating	Extended abstract	Caring/ Application/ Integration	Empowering undergraduate students in RCR is likely to result in a continuous engagement in responsible academic practices throughout students’ academic life and the development of new ethical skills and competences.

For the early career researchers, more specifically PhD students, three private online courses have been developed that focus on themes of supervision and collaboration, on data-related issues of RCR and on authorship and publication. Learning goals were initially developed using Bloom’s taxonomy ( Table 6).

**Table 6.** INTEGRITY early-career researcher learning objectives.

Learning objective	Bloom’s revised Taxonomy	SOLO Taxonomy	Taxonomy of Significant Learning
1. Students are able to explain what values underlie their own research projects	Understanding	Multistructural	Foundational Knowledge/ Integration
2. Students are able to demonstrate what composes a good research practice in their discipline	Applying & Analyse	Relational	Application/ Caring
3. Students can discuss dilemmas in their own research practice	Evaluating	Relational	Human Dimension/ Application/ Integration
4. Students are able to discuss responsibilities and expectations in supervision and mentoring	Evaluating	Relational	Human Dimension/ Foundational Knowledge
5. Students are able to deal with intellectual property and publication issues in a fair manner	Evaluating	Relational	Foundational Knowledge/ Application
6. Students know where to find support in handling issues with third parties	Applying	Relational	Human Dimension/ Learning to Learn/ Caring
7. Students demonstrate the ability to determine authorship order and include acknowledgements when preparing a manuscript	Creating	Relational	Foundational Knowledge/ Application
8. Students know what criteria to use when peer-reviewing work of others	Evaluating	Multistructural	Foundational Knowledge
9. Students are able to distinguish the elements in a data management plan and how to find support to improve it	Understanding & Applying	Relational	Foundational Knowledge/ Integration/ Human Dimension

### VIRT²UE learning objectives

The training developed in VIRT²UE targets researchers ( Table 7) and research integrity trainers ( Table 8). Pedagogical underpinnings involve fostering virtues, stimulating a common ethos of science, learning by doing, and applying a learner-centred approach. Especially the learner-centred approach appears to align with the high-order learning objectives but also learning-by-doing is harnessed to facilitate application and creation.

**Table 7.** VIRT²UE researcher learning objectives.

Learning objective	Bloom’s revised Taxonomy	SOLO Taxonomy	Taxonomy of Significant Learning	Pedagogical underpinnings
1. Identify and apply the core principles and practice of the European Code of Conduct for Research Integrity	Remembering and applying	Relational	Foundational Knowledge/ Application/ Learning to learn	**Stimulating a common ethos of science** This objective sets the ground for reflection on and, development of, a common ethos of science by focussing on the knowledge and application of the core principles of good scientific practice. During the training, participants first learn about the principles through individual learning (via the e-learning modules) and then reflect on them during the participatory exercises by applying them to real or fictional research integrity cases.
2. Understand core virtue ethics concepts and terms and relate these to RI	Understanding and analysing	Relational	Foundational Knowledge/ Integration/ Learning to learn/ Application	**Teaching on virtues, learning by doing** This objective reflects the focus on virtue ethics of the training. Participants first get acquainted with the concepts and theory of virtue ethics and then are asked to use these concepts to analyze and reflect on research practices and on their own research integrity cases. A learning by doing approach is fostered through group reflections, where participants, through dialogue with others, develop an understanding of what virtues mean in a particular context, and how they can be used to analyse real questions or to guide and support good research practices.
3. Engage in case-based and experiential exercises aimed at fostering reflection on virtues	Applying	Relational	Learning to learn/ Human Dimension/ Caring	**Learning by doing, teaching on virtue** By fostering a learning by doing approach, participants learn how to engage in dialogue with others in case-based reflections on the value of virtues for the practice of good science.
4. Recognise RI dilemmas in practice and reflect on professional responsibilities and virtues when resolving dilemmas.	Analysing and evaluating	Relational	Foundational Knowledge/ Human Dimension/ Learning to Learn/ Application	**Learner-centred approach, stimulating a common ethics of science** Through participatory exercises, participants reflect on their own practice and learn how to recognise the research integrity questions and dilemmas they themselves experience. This learner centred approach supports participants in building bridges between theory and practice and stimulates reflection on a common ethos of science by encouraging reflection, in dialogue with others, on the importance of virtues and professional responsibilities in addressing research integrity issues.

**Table 8.** VIRT ^2^UE trainer learning objectives.

Learning objective	Bloom’s revised Taxonomy	SOLO Taxonomy	Taxonomy of Significant Learning	Pedagogical underpinnings
1. Identify and apply the core principles and recommended good practices of the European Code of Conduct for Research Integrity	Remembering and applying	Relational	Foundational Knowledge/ Application/ Learning to learn/ Integration	**Stimulating a common ethos of science** This objective sets the ground for reflection on and development of common ethos of science by focussing on the knowledge and application of the core principles of good scientific practice. During the training, participants first learn about the principles through individual learning (via the e-learning modules) and then reflect on them during the participatory exercises by applying them to real or fictional research integrity cases.
2. Understand core virtue ethics concepts and terms, and relate virtue ethics to research integrity	Understanding and analysing	Relational	Foundational Knowledge/ Integration/ Application/ Caring/ Learning to Learn	**Teaching on virtues and learning by doing** This objective reflects the focus on virtue ethics of the training. Participants first get acquainted with the concepts and theory of virtue ethics and then are asked to use these concepts to analyze and reflect on research practices and on their own research integrity cases. A learning by doing approach is fostered through group reflections, where participants, through dialogue with others, develop an understanding of what virtues mean in a particular context, and how they can be used to analyze real questions or to guide and support good research practices.
3. Facilitate case-based and experiential exercises aimed at fostering reflection on virtues	Creating	Extended abstract	Learning to learn/ Human Dimension/ Caring	**Learning by doing and teaching on virtue** By fostering a learning by doing approach, to achieve this objective, participants learn how to facilitate case-based reflections by experiencing the exercises first as participants, and then by practicing the facilitation of the same reflection in others. Moreover, because the focus of these exercises is to reflect on the importance of virtues for supporting good practices of science, this learning objective is in line with a virtue approach.
4. Critically reflect on experiences of teaching	Evaluating	Relational	Learning to learn/ Caring/ Human Dimension	**Learner-centred approach, learning by doing** As an element of their training experience, participants are asked to reflect on their experience with the exercises (through self-reflection forms) paying attention to their own development as trainers. This supports participants in reflecting on their personal teaching skills and style. The reflection on one's teaching experience is also fostered through group reflection, where participants share their own experiences and get peer support and feedback from other participants and trainers. This focus on self-reflection and peer to peer feedback fosters a learner centred and learning by doing approach.
5. Adapt teaching approaches based on the target group characteristics	Creating	Extended abstract	Learning to Learn/ Caring	**Learner-centred approach** Participants reflect on their teaching context and target audience to be able to embed the training materials in their own teaching practice. This attention to follow up and to the trainees’ teaching needs and context is in line with a learner centred approach.

### ENERI learning objectives

The training developed in ENERI targets members, old and new, of RI offices and RE boards and other bodies, in which RE and RI expertise is required ( Table 9). Constructivist ideas of building on prior knowledge and emphasising the relevance for the learner permeate the learning objectives of the ENERI training.

**Table 9.** ENERI expert learning objectives.

Learning objective	Bloom’s revised Taxonomy	SOLO Taxonomy	Taxonomy of Significant Learning	Pedagogical underpinnings
1. Gain overview of main features of research integrity as perceived in today’s society (i.e., based on ALLEA Code of Conduct)	Understanding	Multistructural	Foundational Knowledge	The relevance of **prior knowledge** is acknowledged in constructivist notions of learning. In the case of experts as learners, this involves updating of prior knowledge to current state-of-the-art.
2. Update familiarity with and review latest changes or modifications to relevant policies, regulations, guidelines, and procedures (global, EU, national) related to research integrity/research ethics and responsible conduct in research	Understanding	Multistructural	Foundational Knowledge	The relevance of **prior knowledge** is acknowledged in constructivist notions of learning. In the case of experts as learners, this involves updating of prior knowledge to current state-of-the-art.
3. Conceptualize the roles of a research integrity board member/integrity officer/research ethics committee member, and the boundaries of those roles	Applying and analysing	Relational	Application/ Integration/ Learning to learn	Learning is meaningful when it **connects to relevant prior knowledge**. Case-based learning supports reflection on own role.
4. Identify key documents including policies, regulations, guidelines and procedures (global, EU, national) as well as networks and other resources related to research ethics, integrity and responsible conduct in research	Remembering and understanding	Multistructural	Foundational Knowledge	Learning is meaningful when it **connects to relevant prior knowledge**. Case-based learning supports reflection on own role.
5. Understand the nature of questions or issues that research integrity advisory boards and ombudspersons/ officers/ethics committees deal with, and embrace the complexities of these issues	Understanding, analysing and evaluating	Relational	Foundational knowledge/ Integration/ Caring	The level of **cognitive complexity gradually increases** by pulling objectives 1-4 together towards exercising judgement necessary in complex situations. Case-based exercises of ethically challenging nature support learning.
6. Identify, develop, and apply appropriate strategies for integrity/research ethics board and committee members for advising on ethical issues/research integrity to build a culture of integrity in research communities, and develop mentorship qualities	Applying, analysing and creating	Extended abstract	Application/ Integration/ Human Dimension/ Caring	Builds on objectives 1-5, especially 3 and 5. E **ncourages to apply beyond current domain**. Case-based exercises of ethically challenging nature support learning. The **level of cognitive complexity gradually increases**.
7. Strengthen networks to bring emerging issues to the attention of academics, policy makers, financing agencies and other key stakeholders. and share experiences and develop good practices in emerging topics	Applying, evaluating and creating	Extended abstract	Caring/ Human Dimension/ Integration	Pulls together prior objectives, **encourages to apply beyond current practice.**

### Combined results

**Figure 1.**
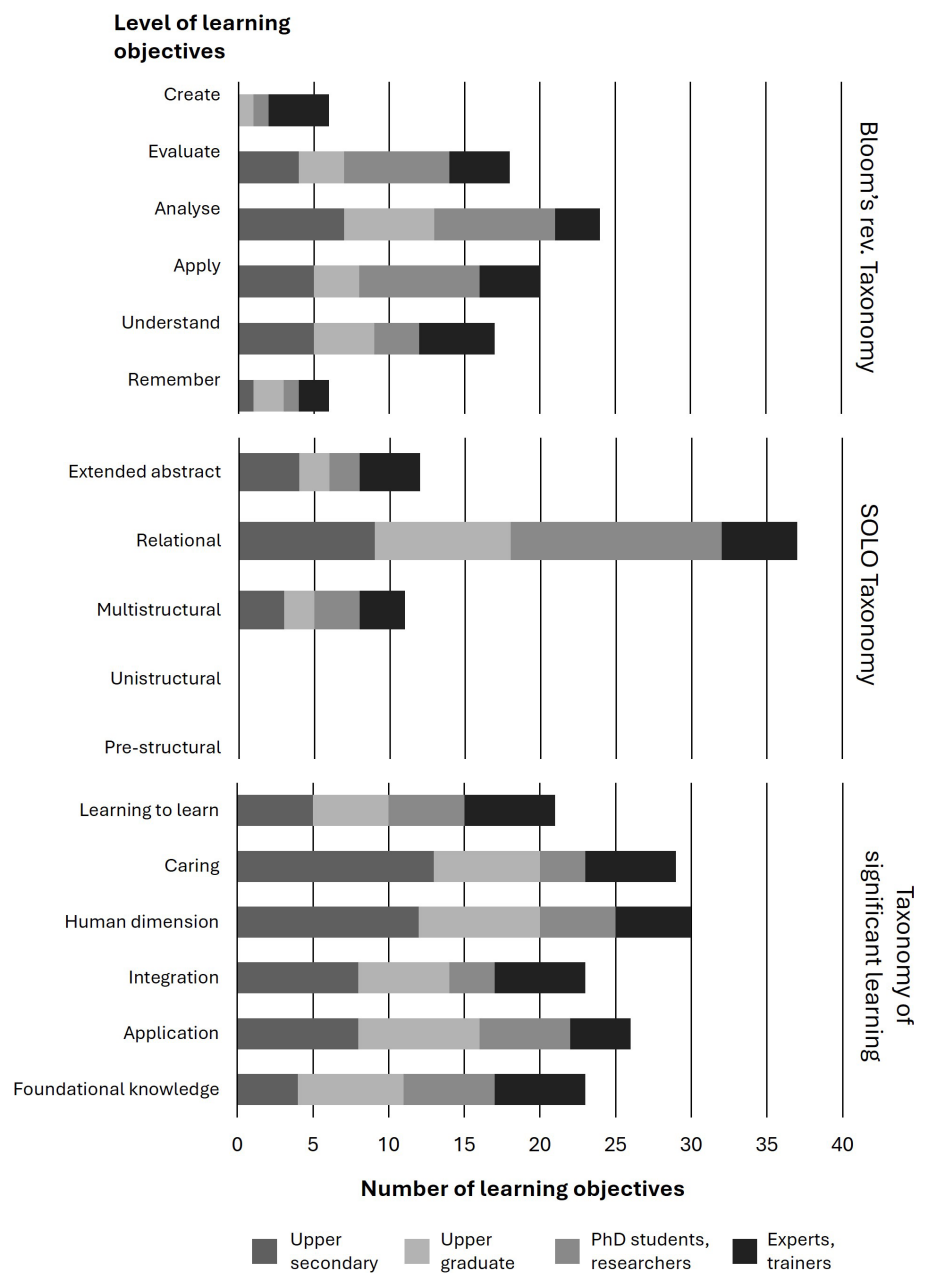
Summary of the frequencies of learning objectives at various training levels.

The analysis shows that there is an emphasis on mid to higher level learning objectives (applying, analysing, evaluating in Blooms’; relational level in SOLO), while the highest levels; creating (Bloom’s) and extended abstract (SOLO), do not feature as frequently ( Figure 1).

**Figure 2a–c.**
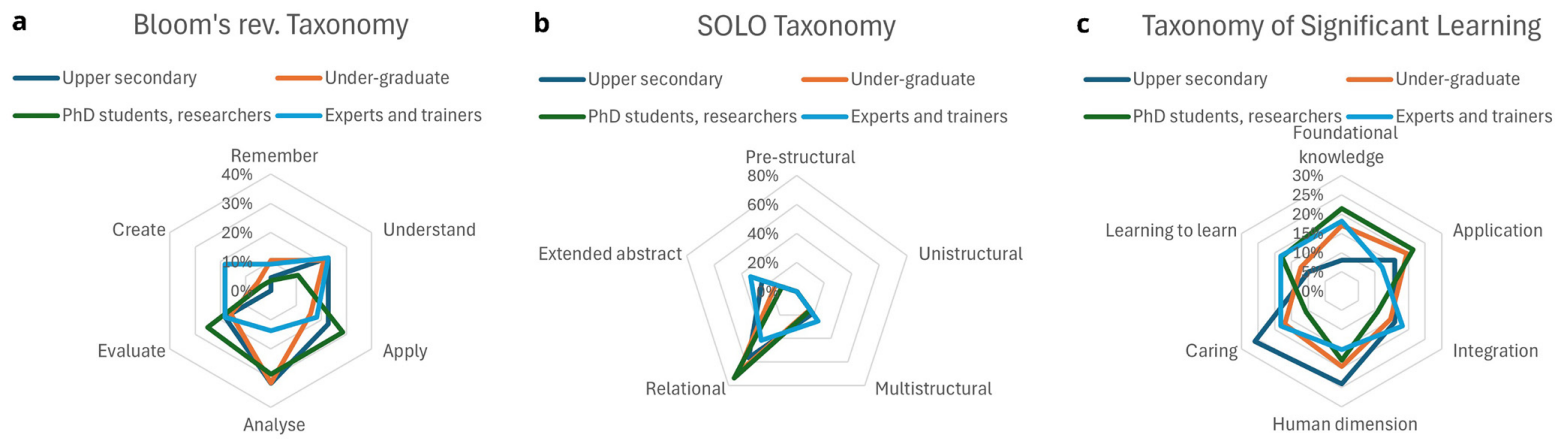
Percentages of learning objectives per training level dedicated to the dimensions of Bloom’s rev. Taxonomy ( **a**), SOLO Taxonomy ( **b**) and Taxonomy of Significant Learning ( **c**).

It is important that there are higher order learning objectives also for training at basic levels. It is a positive result that this is the case among the analysed trainings ( Figure 2a). For none of the target groups the level of Remembering is prominent. Experts and Trainers are also challenged to Create. For PhD-candidates and researchers (the target groups that have to bring the lessons learnt to practice in their research and are role-models to upper-secondary and under-graduate students) the focus is on Application, Analysis, and Evaluation ( Figure 2a). It is also noteworthy that the lower levels in the SOLO Taxonomy; pre- and unistructural levels of understanding, are not present indicating a focus towards higher-order learning. Figure 2b shows that the Relational level is most prevalent in the trainings. Experts and trainers also significantly address Multistructural and Extended abstract.

All categories of the Taxonomy of Significant Learning feature in all trainings suggesting that the trainings foster caring and the human dimension ( Figure 2c). Nevertheless, there are some significant differences. The main load of the trainings’ learning objectives for the upper secondary level are placed at Caring, Human dimension and Application. At graduate and undergraduate level, gaining Foundational knowledge and Application are key parts of the training program and at graduate level, for PhD candidates and researchers, Learning-to-learn is added to that. Application is less frequent for experts and trainers, while all other aspects of Significant Learning remain almost equally present in their training.

**Figure 3.**
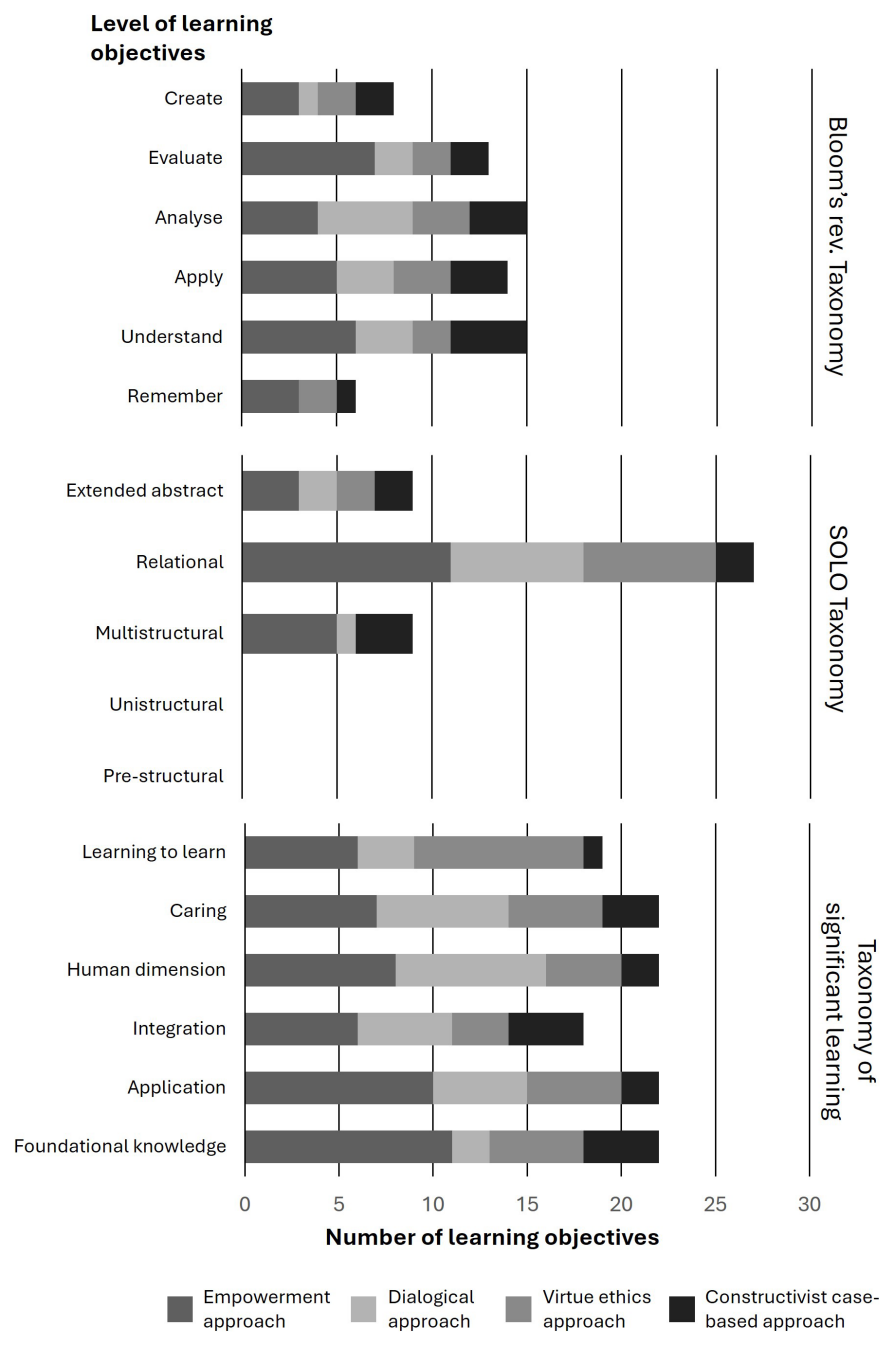
Summary of the levels of understanding supported by the four training approaches.

**Figure 4a–c.**
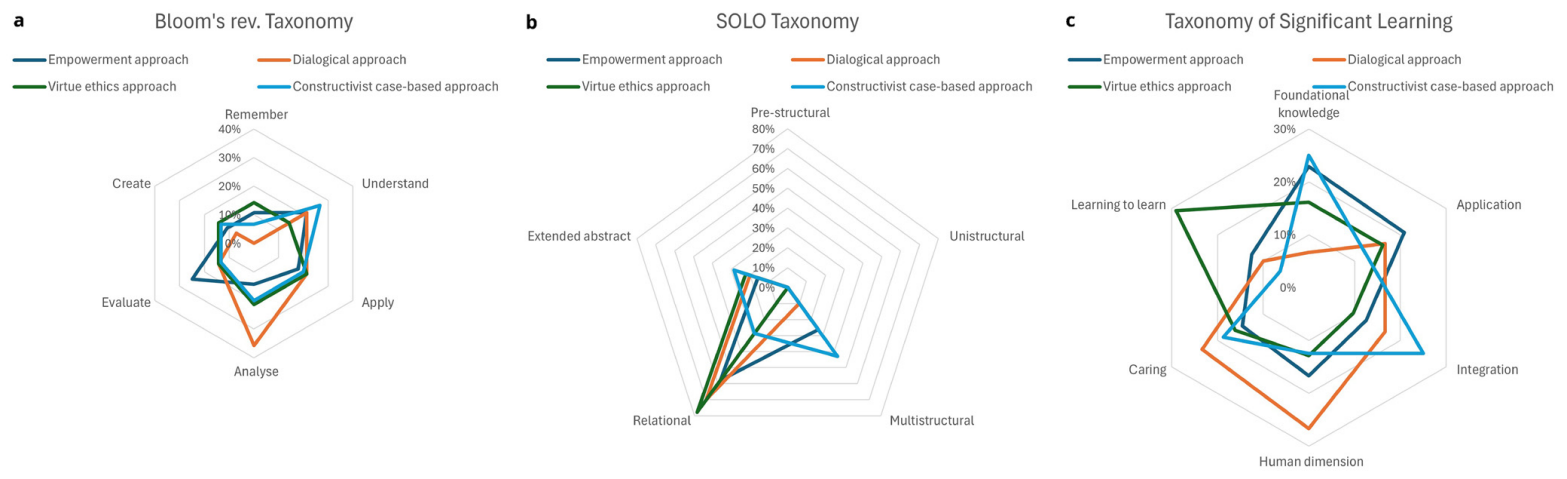
Summary of the levels of understanding supported by the four training approaches according to three taxonomies.

There is a relatively balanced application of different level learning objectives across the pedagogical approaches ( Figure 3). The dialogical approach features heavily learning objectives at the analytical level (Bloom’s taxonomy) ( Figure 4a). Relational knowledge (SOLO taxonomy) features highly in all but the constructivist case-based approach ( Figure 4b). Foundational knowledge (Significant learning taxonomy) features relatively frequently among all approaches except for the dialogical approach, in which the accumulation of objectives lands on the human dimension and caring ( Figure 4c). These dimensions appear highly relevant considering the nature of dialogic learning. Similarly, learning-to-learn features relatively frequently among the objectives of VIRT²UE. Learning competencies involve reflection, which was also strongly present in the VIRT²UE training. While understanding and applying are frequently reoccurring, this appears to be seen as a prerequisite for further learning to take place. This is particularly evident in the empowerment, dialogical and constructivist case-based approach. In the virtue-ethics based approach, understanding is slightly less prominent, but this may be because the division of learning objectives levels out a bit more over all levels of Bloom’s revised Taxonomy.

We do not regard the levels of the learning objectives to be pertinent features of specific approaches, but merely demonstrate how each approach can and indeed has considered the various taxonomic levels. There are also differences in how the various levels are considered. For example, the empowerment approach has a stronger emphasis on objectives aimed at facilitating learners’ evaluative competencies than the other approaches. The dialogical approach emerges as successful in incorporating objectives aimed at facilitating analysis.

## Conclusions

The four RI trainings are underpinned by either a constructivist or a socio-constructivist understanding of learning; they implement activating and engaging learning activities, sometimes in collaboration with others, and they have elaborated objectives regarding learning. They all, irrespective of pedagogical underpinnings, emphasise high-order objectives. The highest level, according to both Bloom’s and SOLO Taxonomy, is not that frequent, but it is present in all four pedagogical approaches and for all training target groups. Relational understanding - the second highest level according to SOLO – features frequently. Similarly, upper mid-level objectives according to Bloom’s Taxonomy are also relatively frequent. This suggests that learning objectives manage to convey elaborate ideas of the level of understanding (as viewed through SOLO) sought through the trainings but are slightly less elaborate on the intentions of what learners are expected to do with the knowledge (Bloom’s). The Taxonomy of Significant Learning adds value by making objectives related to the human dimension, caring and learning-to-learn visible. Such learning objectives bring forth affective and reflective objectives, which are relevant to the learning of integrity and ethics but may not always be emphasised as intended learning outcomes. Some ethical decision-making models (e.g., Bebeau *et al*., 1999; Butterfield *et al*., 2000; Haidt, 2001) emphasise the importance of emotions as a complement to a rational cognition and decision-making.

Based on the results, we have identified the following implications for training design and recommendations for training developers:

- The trainings analysed are based on diverse and elaborated pedagogical conceptions, which are operationalised through explicitly spelled-out learning objectives. The analysis suggests that taxonomies are applicable to various pedagogical underpinnings and can help trainers to communicate the objectives of training and learners to relate their expectations to the objectives.
- Training developers have a variety of taxonomies at their disposal, and less commonly applied taxonomies in the context of integrity training, such as the Taxonomy of Significant Learning ( Dee Fink, 2003) can help to identify relevant learning objectives both for trainers as well as learners.
- When developing training, it is advisable to pay more attention to learning objectives dedicated to foster the highest levels of understanding and the meaningful use of knowledge gained.
- When employing taxonomies to set learning objectives and assess learning, it is fundamental that the training facilitates higher-order thinking, that is, while the training may be targeted at various groups, such as high-school students or senior researchers, high-order thinking can always be stimulated at an appropriate level, as shown by the analysis of the trainings.
- Research cultures and disciplinary differences are generally not explicitly spelled out at the level of learning objectives. There is a risk that both trainers and participants make assumptions about cultures and contexts and spelling these out may prevent misalignment of expectations.
- The trainings address learning objectives that aim to provide the learners with the skills and knowledge that enable them, and an attitude that motivates them, to navigate difficult situations they may encounter while performing research. This implicitly tells us the training developers of each of these trainings consider the research environment to be challenging and not automatically fostering responsible research practices. Therefore, we need trainings such as these four training programs.
- Moreover, it is necessary that a large number of researchers and others involved in research participate in such training, so that they can together navigate dilemmas in their research endeavour and create a responsible research environment. One may think of it as creating ‘herd immunity’ by training many stakeholders in research. The trainings use dialogical methods and group learning, which contribute to the shared responsibility to foster a responsible research environment. Additionally, by training trainers and experts, the VIRT²UE and ENERI trainings also contribute to building a culture of integrity.

There are limitations in this research. A limitation is that we have been ourselves involved in these projects and have engaged in this analysis in a self-reflective manner, however, it is possible that we are biased in how we see our projects. The fact that we have worked collaboratively and in transparent manner is likely to alleviate some of the potential bias, but we cannot entirely rule out interpretations made in favour of our own views. Therefore, applying also another approach in two analyses (SOLO, Significant Learning) utilising the expertise of an individual external to the projects may have further decreased bias. Furthermore, the selection of training is limited to those developed within the frame of four large-scale European projects funded under the European Union’s Horizon 2020 research and innovation programme. We recognise that there are other projects, which have or are currently developing training materials for research ethics and integrity that have not been included in this analysis. The inclusion of a broader range of training may have broadened the scope of the results. Indeed, a topic for further research involves the analysis of how the conceptions of teaching and learning RI have evolved across European Union funding programs reflecting various development needs at various points of time.

## Declarations

### Ethics and consent

Ethical approval and consent were not required.

## Data Availability

The data consists of publicised learning objectives of the trainings and materials produced in four European Union-funded projects. The learning objectives can be found on the websites of the four projects: Path2Integrity: https://learning-p2i.eu/ INTEGRITY: https://embassy.science/wiki/Guide:39eb092a-b4be-41a3-a070-872cbfbcc6ca VIRT²UE: https://embassy.science/wiki/Guide:Bbe860a3-56a9-45f7-b787-031689729e52 ENERI: https://classroom.eneri.eu/
