# Supplementary material for: Fostering Research Integrity through Training: The training materials of four EU-funded projects through the lens of pedagogical underpinnings and three taxonomies of learning
Source: Open Res Eur. 2025 Sep 26;5:301. [Version 1] doi: 10.12688/openreseurope.20826.1 (PMC12640486; doi:10.12688/openreseurope.20826.1)
Supplement: Peer review history

# Reviewer report: Reviewer response for version 1

**DOI:** 10.21956/openreseurope.22532.r62502

**Published:** 2025-11-22

**Authors:** Maha Emad Ibrahim

**Affiliations:** 1 Suez Canal University, Ismailia, Ismailia Governorate, Egypt

This article analyzes 4 EU projects that focus on teaching research integrity using 4 different methodological approaches. The analysis is qualitative in nature and utilizes 3 types of learning taxonomies. 

 I find the article well written, the conclusions are supported by the analysis. The recommendations are insightful hopefully giving the Horizon 2020 a roadmap for future RI projects.

 I would recommend future research to address how these learning objectives translate to results for learners based on data provided after project implementation via M&E or other oversight data. This will provide a results-based analysis that when linked with this one will give an impression of how these learning objectives operate on the ground.

If applicable, is the statistical analysis and its interpretation appropriate?

Not applicable

Is the study design appropriate and is the work technically sound?

Yes

Is the work clearly and accurately presented and does it engage with the current literature?

Yes

Are the conclusions drawn adequately supported by the results?

Yes

Are sufficient details of methods and analysis provided to allow replication by others?

Yes

Are all the source data and materials underlying the results available?

Yes

Reviewer Expertise:

Research Ethics, including Research integrity

I confirm that I have read this submission and believe that I have an appropriate level of expertise to confirm that it is of an acceptable scientific standard.

# Reviewer report: Reviewer response for version 1

**DOI:** 10.21956/openreseurope.22532.r62501

**Published:** 2025-10-31

**Authors:** Sarah Elaine Eaton

**Affiliations:** 1 University of Calgary, Calgary, Canada

The researchers analyze materials and training content from four EU-funded projects focused on research integrity. The data for this project are the training materials, which were analyzed from a dialogical, empowerment, virtue ethics and constructivist case-based approach. Results showed that the training materials adopted either a constructivist or socio-constructivist approach to learning and emphasize higher-order learning objectives. Recommendations for developing training materials are included based on results. The findings from this research could have significance for the future development of research integrity training materials.

If applicable, is the statistical analysis and its interpretation appropriate?

Not applicable

Is the study design appropriate and is the work technically sound?

Yes

Is the work clearly and accurately presented and does it engage with the current literature?

Yes

Are the conclusions drawn adequately supported by the results?

Yes

Are sufficient details of methods and analysis provided to allow replication by others?

Yes

Are all the source data and materials underlying the results available?

Yes

Reviewer Expertise:

academic integrity, research integrity, research ethics, ethics

I confirm that I have read this submission and believe that I have an appropriate level of expertise to confirm that it is of an acceptable scientific standard.

## References

[1] : Fostering Research Integrity through Training: The training materials of four EU-funded projects through the lens of pedagogical underpinnings and three taxonomies of learning. *Open Research Europe* .2025;5: 10.12688/openreseurope.20826.1 10.12688/openreseurope.20826.1 40386579
